# Notes on Myotomy and Tenotomy

**Published:** 1841-11-13

**Authors:** Reynell Coates


### ORIGINAL COMMUNICATION.

### NOTES ON MYOTOMY AND TENOTOMY.

BY REYNELL COATES, M. D.

To the Editors of the Medical Examiner.

GENTLEMEN,—Having become more intimately connected with your Journal than was expected when this series of essays was commenced, the unforeseen length to which it has been extended is not the only reason for bringing it to a conclusion for the present. It requires a greater devotion of time than can be spared from the demands of other duties at this season of the year. Deferring, then, till some more suitable occasion, the consideration of the construction of the various forms of apparatus, and the contemplated remarks on other applications of Myotomy to the cure of deformities or other defects of motion, (most of which, fortunately for humanity, *have condemned themselves*,) I propose to add a few words on two or three points to which allusion has been made in the preceding papers, and finish with two or three general conclusions.

In the contest between the "*machinists*" and the "tenotomists,"—between the advocates of the simple mode of treatment, and the more complex—the principal grounds of dispute relate to the relative amount of time expended and the degree of pain suffered during the treatment in either case.

It is certainly surprising, at a first glance, that, in the only tolerably well reported series of cases in which the mechanicnl treatment, so called, has been exclusively employed, the time occupied in effecting what is termed a cure, is not materially, if at all, longer than that represented to be occupied by the tenotomists,—I allude to the cases contained in the papers of Dr. Heber Chase in the American Journnl of the Medical Sciences, for January, 1841. The general accuracy of these cases I have been at the pains to substantiate by personal examination. This, however, becomes less surprising when we consider that the necessity for mechanical after-treatment is not removed by tendon cutting, and that in this after-treatment the mechanical forces act at a material disadvantage in consequence of the preceding operation. But I hold it to be undeniably a misfortune when the cure, so called, is effected *rapidly*. Where bones are to be remodelled, ligaments permanently elongated or shortened, original joints changed in their form and position, and sometimes newly formed joints to be destroyed and obliterated, it appears plain to me—and observation bears out the conclusion,—that the more rapidly the foot is brought into the condition styled *a cure*, the more considerable and intractible must be the permanent deformity. It is even possible for an awkwardly constructed apparatus to produce undue abduction, or, in other words, a commencement of a valgus of the anterior part of the foot while the ossa calcis and astragalus continue in the attitude characteristic of the first stage of varus! It is more than probable, from the description of the celebrated case of M. Delpech, as given before the Royal Academy, by M. Bouvier, that this very deformity was produced after tenotomy by that unfortunate surgeon. These remarks will be sufficient, I think, to convince any cautious practitioner that the rapidity of action in such cases, which looks so *brilliant* before a class, and proves so captivating to the vulgar and the ignorant, is undesirable for the patient,—if it be not an evidence of decidedly bad surgery,—at least in the case of children over six years of age. Very slight deformities of the tarsus are often productive of most serious inconvenience. I have been consulted within a few days in the case of a gentleman in extensive business who has been rendered nearly powerless in the transaction of ordinary affairs by a slight adduction of the foot, the precise nature of which cannot be accurately ascertained. It was supposed to be the result of a fracture of the tibia involving the ankle joint, accompanied by an injury of the astragalus and the os calcis which escaped attention at the time of the accident. The present condition of the tarsus—some months after the injury—displays a not very explicable subluxation of these last named bones, accompanied by a considerable deposit of callus between the outer sides of the astragalo-calcanean and the astragalo-fibular connections. The consequences of the deformity are such as to retain the foot in a slight state of adduction, interfering absolutely with the power of the muscles to carry the foot outwards, and compelling the patient to walk somewhat upon the outside of the sole. The inconveniences resulting are such as induce him to submit to a treatment requiring many months of time and very considerable expense with the view to promote facility in the transaction of business.

There can be no doubt, in the present state of physiological investigation, that the osseous tissue, in common with all other tissues, is subject to an almost indefinite series of changes under the action of moderate forces, if properly regulated, so as to remove the most serious deformities, if the surgeon be only contented to employ a proper amount of time in the effort.

If we needed positive testimony on this point, it would be found in the history of a still pendent case now under treatment in this city, in which an adult has been subjected to a gradual absorption of the outer side of the femero-tibial articulation. From the pressure of the exterior condyle of the thigh bone, the exterior depression of the tibia was modified by absorption, in consequence of a deficient deposit of ossific matter, until the leg became almost incapable of maintaining the weight of the body. The interior lateral ligament of the knee joint was violently extended, and when the knees were placed in apposition, the legs made with each other an angle of not less than twenty-five degrees. The feet were brought flatly to the ground, by a modification of the form of the ankle-joint. The deformity had been rapidly increasing for months. Yet the application of an apparatus embracing the pelvis, and acting upon the legs in such a manner as to approximate the feet without preventing the motion of the ankle and the knee, is rapidly restoring the legs to their straight position.—Whether this is a mere temporary or a permanent advantage, remains to be determined; but the result will be duly reported.

Dr. Chase has been experimenting largely, of late, on the power of mechanical forces in extending joints affected with false anchylosis, and the result, which will appear in the next number of the American Journal of the Medical Sciences, and which I have been permitted already to peruse in the manuscript, adds weight to the position that the most formidable deformities of the osseous apparatus cannot resist the long continued action of rightly directed mechanical forces. That the bones of children are capable of being remodelled, for good or evil purpose, by such means, is well known—that those of adults, even to the latest age, are capable of undergoing similar changes, it needed not these experiments to prove.—The bandy-leg and the knock-knee, are complaints curable without the aid of tenotomy,—not in youths only, but in adults. The only question, in such cases, is whether the advantage to be gained by the cure will compensate the time and expense of the treatment. This, indeed, should be the conclusion of every thinking man when he becomes aware of the fact that the bones of the chest are rapidly absorbed under the pressure of a thoracic aneurism—that those of the head are enlarged or contracted in order that they may be adapted to the varying dimensions of the brain, *up to the latest period of life*—and that the same causes which produce club-foot in the adult, by being reversed, become capable of undoing the mischief they have done. In fine, it may be laid down as a settled law, that there is no definite limit to the changes of form which may be produced by steady and moderate action upon a bone or a joint, other than what necessarily results from the time necessarily expended in the attempt, and the ability of the soft parts to endure pressure. Nor is the production of considerable pain at all necessary in accomplishing such changes, if the attempt be made with moderation; with due regard to the broad differences observable in different parts of the body in relation to their capacity for the endurance of pressure; and the exercise of proper caution in the avoidance of embarrassments to the circulation.

Even in the present day, then, though most of the apparatus for club-foot is so imperfectly adapted to the purpose in view, the degree of success following the purely mechanical treatment of that disease is sufficient to render tenotomy, under ordinary circumstances, an operation of exceedingly questionable propriety; and such I seriously believe to be, at present, the prevailing opinion of the profession in this city. When the apparatus becomes matured by further study and improvement, this opinion will unquestionably gain ground. Much of the present imperfection of machinery results from an improper desire to enable the patient to walk about during the whole course of the treatment. During the first stage of the treatment of a confirmed varus of the third degree, *it does not appear possible properly to adapt to the case a suitable instrument*, without forbidding all attempts to walk. During the second stage, *it is possible*, by means of complex araangements, but it is very doubtful whether it is *desirable*. In the first degree of varus, whether resulting from the treatment of the worse grades, or an original condition of the parts, the freedom of locomotion should not be restricted; for, in such cases, as in the *pes equinus*, the attempt to walk rather aids than interferes with the operation of the instrument. But, it should always be remembered that it is rather the *permanency* than the *force* of action that must be depended on to produce the most beneficial results.

I have spoken, in a former article, of the slight adduction of the os calcis that persists in all the cases yet examined after the cure—so called—is complete, whether tenotomy has been performed or not. For the removal of this by no means insignificant relic of the deformity, I know of no tolerably well adapted instrument. The shoes provided with springs for this purpose are very objectionable, on grounds already pointed out; and, at best, shoes of any kind are extremely inconvenient contrivances where nicety and certainty of action are required. I am about to undertake the improvement of this part of the treatment, and, if successful, will be happy to make known the result.

In varus, the knee-joint is always more or less modified in form, and in old and bad cases, the hip-joint also undergoes corresponding changes. In the knee, this modification appears to consist in the elongation of the lateral and crucial ligaments, together with a partial absorption of the cartilages, permitting the leg to rotate on the thigh, in order to allow the toes to be pointed unduly inward. This produces a very undue degree of mobility in the joint; and, after the nearly complete reduction of the foot to its proper position, mechanical support of a nature calculated to prevent rotation until the joint recovers its firmness, should never be neglected. The character of the changes of form in the acetabulum, and, it may be, in the neck of the os femoris also, is more occult; but the purpose and result of the alteration are precisely similar to those observed in the knee. The application of instruments for the correction of these deformities of the hip-joint is difficult, expensive, and exceedingly annoying to the patient. Fortunately, voluntary efforts and the exercise of constant care in rotating the toes outward may render such apparatus unnecessary.

The principal differences important to therapeutics between the varus and the pes equinus are these: The latter is very seldom congenital, and it is very generally spasmodic in its origin. The nature of the causes that usually produce it is such as to warrant the conclusion that nervous action,—and *that*, commonly of a reflex character,—is the origin of the muscular retraction. The nervous condition, be it merely functional or be it structural, is temporary. If structural, it is liable to end in paralysis. But the muscular *effects* of this condition are persistent; and lead more frequently to the chondroid transformation of tissue than those occurring in any other form of club-foot. In varus, on the contrary, the nervous causes of contraction, if they have been present at any time, have usually consummated their effect before the period of birth; and, the changes of tissue are rather the result of the malposition of the parts than the consequence of the original nervous affection. It follows from these remarks, that tenotomy is more frequently requisite in the pes equinus than it is in varus.

The general wasting of the limb observed in most cases of club-foot, is evidently as frequently the result of the inactivity of the member as of any primary affection of the nerves producing a vice of nutrition. At least, this is obviously true in most instances occurring after birth, and it is a fair inference that the same rule will apply to the majority of congenital cases. It is noticed that in club-foot occurring in early life, and affecting one side only, the corresponding limb is less developed in longitude as well as in circumference: and it would be a curious and useful investigation to ascertain how far the cure of the deformity of the foot is capable of removing this evil in the progress of years.

Having now brought to a conclusion this somewhat desultory series of remarks, it will be well to state a few general conclusions which appear to me to be legitimately deduced from their whole tenor.

1st. When club-foot is the result of spasmodic action, no attempt should be made to remove the deformity either by mechanical force or by tenotomy, until the spasmodic action is exhausted naturally, or has been removed by general or local measures of a totally different character.

2d. When the contracted muscles have undergone a chondroid transformation, tenotomy is preferable to the exclusive use of mechanical extension.

3d. In all other cases, the mechanical extension should take precedence of tenotomy, and the latter operation, if performed at all, should never be attempted in patients whose pecuniary and social condition admit of reasonable delay, until all other means have been faithfully tried without avail.

4th. The mere degree of deformity never precludes the possibility of cure without the aid of the knife.

5th. Tenotomy rather enhances than diminishes the difficulty of effecting a perfect cure by mechanical means.

6th. The foot may be restored *to a useful, but not to a natural position* in pes equinus, more speedily by a resort to tenotomy than by the effects of machinery alone.

7th. In confirmed varus of the second and third degrees, it is not desirable that the apparatus should permit the patient to walk; and it is proper that the instrument should be extended to the thigh in order that action of the forces should be rendered as direct as possible.

8th. No degree of deformity unattended with true anchylosis of the tarsal bones, ought to be regarded as absolutely incurable by mechanical means alone.

It is proper to remark, in conclusion, that the necessities of the poor *may* require a resort to tenotomy in order to reduce a foot to an attitude consistent with the performance of labour in the shortest possible time; but this advantage must be gained at the expense of some considerable degree of persistent deforformity; and there is every reason to believe that, notwithstanding the evidence of the tenotomists to the contrary, there must generally be a permanent loss of power in the muscles of which the tendons are divided.

